# American Academy of Dental Science

**Published:** 1899-09

**Authors:** 

**Affiliations:** American Academy of Dental Science


### AMERICAN ACADEMY OF DENTAL SCIENCE.

The regular monthly meeting of the American Academy of Dental Science was held at Young's Hotel, Wednesday evening, March 1, 1899, at six o'clock.

A paper was read by George R. Southwick, M.D., of Boston, entitled "The Lesions of Syphilis of the Mouth."

(For Dr. Southwick's paper, see page 576.)

### DISCUSSION.

*Dr. Eames.*—I wish to express my thanks to the essayist for responding to my request to present this subject, because I think it is an extremely important one. These extensive sores on the face, which are exposed to view, are not necessarily those which will lead us into trouble, because they are unsightly and we are put on our guard at once. The large lesions are warnings in themselves to us, but many times more important, it seems to me, are those cases of syphilis the lesions of which are not exposed to view, but are hidden behind the soft palate or in the nose; and from these sores the saliva in any part of the mouth may become infected, and thus convey the virus just as truly as if the lesions were in sight. The only safe practice, therefore, is to be always on guard, not wounding the mucous membrane; and protecting any raw surface on our hands, and using great care in sterilizing our instruments.

*Dr. Potter.*—That point I would like to ask Dr. Southwick about,—the point that has just been referred to in regard to the infection by saliva, where there are no lesions in the mouth. I was under the impression that if there were no secreting lesions, the saliva of itself was not infectious.

*Dr. Southwick.*—I should not like to express a positive opinion, but if I should happen to have a hang-nail I would be very careful about putting my fingers into the mouth of a patient where I had any reason to suspect syphilis. There might be a lesion of the mucous membrane somewhere, although not in sight; there might be an ulcer on the pharynx, in the larynx, or in the posterior nares, and under these circumstances the saliva becomes at once infectious. Could we be absolutely sure that all syphilitic lesions were excluded from the mouth, nose, and throat, I should say that it was doubtful if the saliva would be infectious.

*Dr. Werner.*—Would the saliva of a person that had evidently had syphilis and was apparently cured still be infectious? I have in mind a young man, perhaps twenty-eight or thirty, whose teeth look like those of an old man. There are evidences of old scars on the mucous membrane where it joins the necks of the teeth, and the mark of an old fissure running up between the incisors, but he is better, and as he expressed it, "I am a new man."

*Dr. Southwick.*—One should be very careful in operating on such a person, because such a patient might have a hidden lesion of the mucous membrane and be infectious. If we could be absolutely sure that there was no lesion about the mouth or adjacent parts, then the infection can be reasonably excluded. We know that the seminal fluid of a syphilitic man does not infect the woman. She will be likely to get it by contact with some lesion, but the mere deposit of the seminal fluid on the mucous membrane does not infect; so if the semen will not do it, I should hardly expect that the saliva alone would infect. Syphilitic persons are apt to have lesions of some sort, even after they are considered to be cured, and if I had occasion to operate on a man whom I knew had had syphilis, I should be rather particular how I got that man's saliva on my hands. It is something which demands infinite care, for he might be all well to-day, and to-morrow there might be a papule which would cause lots of trouble to the operator.

*A Member.*—Do you think, then, that a person can never be said to be entirely cured of syphilis?

*Dr. Southwick.*—It is a serious question whether you can say that a person is completely cured of syphilis and will never have recurrence, and cannot transmit it to his children. It is claimed by the authorities that a person who has been carefully treated until all lesions have disappeared, and who shows no recurrence of secondary symptoms for a period of five years, can be said to be cured, but that is not always true. I can tell you of a case in my practice which will show you the tenacity of the disease even after it is pronounced cured. A certain lady contracted syphilis from her first husband, was treated for it by the best physicians, and declared to be cured. Some years after, a gentleman asked her to marry him. She told him all the facts of the case, and, as he was still anxious to marry her, they consulted some of the best authorities here and in New York. As it was a number of years more than five since there had been any manifestations, they all pronounced her safe from recurrence, and told her that she was absolutely cured. She was married. Her first child was born about two years afterwards, was sickly, poorly nourished, and died in infancy. Another child was born about the fifth or sixth year of her marriage, and, although delicate, there were no positive signs of syphilis. A third child was born about the tenth year of her marriage, and that child showed unmistakable signs of syphilis. The case first came under my notice when called to treat this child for caries of the superior maxilla. The father assured me there was no infection. I curetted and curetted, but could not stop the disease. Finally I gave that youngster some iodide of potassium, and he got well. I then put some pretty straight questions to the parents, and they admitted the facts as I have given them to you, but had said nothing to me about it before, because they had been told that the disease was absolutely cured. The second husband had not had it. The patient had some nasal trouble, with considerable chronic coryza, which may or may not have been caused by syphilis, but it certainly shows the heredity of the disease, although it was in the neighborhood of twelve years from the time when she was pronounced cured by physicians who are considered to be authorities on the disease; so that, personally, I am rather sceptical regarding the absolute cure of syphilis. It is true, there are very many cases in which the disease does not return, but there is always a possibility that at some time of life it may manifest itself.

*Dr. Potter.*—I would like to ask, in regard to the enlargement of the glands, whether they are characteristically enlarged in syphilitic persons or not? I have always associated a round, shot-like feeling under the finger with syphilis.

*Dr. Southwick.*—There is a characteristic feeling to the glands about the genitals when the initial lesion has occurred there. You will find that the glands in the groins have a spindle-shape rather than the round shape, which is characteristic of the ordinary infection of the chancroid poison, but I do not know of any particular type or form of glandular enlargement which occurs about the mouth and is in itself characteristic of syphilitic infection and not seen in other conditions.

*Dr. Taft.*—I would like to ask Dr. Southwick if in the illustrations he has shown us of ulcers in the mouth there were also ulcers in other parts of the body as well as the mouth, and whether he cured each individual ulcer with the inunction he spoke of?

*Dr. Southwick.*—Lesions of the mouth are usually accompanied by cutaneous lesions which may be located on any portion of the body, and other signs of syphilis were present in many of the cases. If the mouth was the seat of the primary infection, we would not have cutaneous manifestations at the same time as the primary sore, but the symptoms of the other stages are liable to manifest themselves in any part of the body. The inunction is a general treatment of all the body, not a single ulcer, and cures all the manifestations of syphilis.

*Dr. Bigelow.*—I would like to ask the essayist if, in the case of a patient who was known to have had syphilis but supposed to be cured, and such a person coming to us for treatment, it would be possible to remove the possibility of infection by any local means, such as mouth-wash?

*Dr. Southwick.*—No; I think the greatest protection would be something which would prevent our coming in contact with the saliva, like thin rubber cots, gloves, such as are used in abdominal surgery, and are thin as tissue paper. Of course those cases are possibilities, but in such important matters we want to exclude every possible chance of infection. Even after I have discontinued the treatment of a syphilitic patient, I recommend them to take occasionally a little mercury or iodide of potassium; I do not mean excessive dosing, but perhaps a little now and then; that is, the patient will be less likely to have a recurrence by so doing. But I should be doubtful about depending upon a wash, for, as I said before, the lesion might be located in the posterior nares, and any gargle or wash would not be likely to reach the seat of infection.

*Dr. Werner.*—And if it did, would it be of any material benefit?

*Dr. Southwick.*—No; the disease is systemic; the sores are persistent and infectious. Cleansing could be only temporary.

*Dr. Fillebrown.*—It seems to me that the rubber dam, thoroughly applied, well adjusted, would be a pretty thorough protection to the operator, the patient, and everybody else.

*Dr. Southwick.*—Yes, after you have succeeded in adjusting it. If I were a dentist I should keep on hand a supply of rubber cots, which are as thin as tissue paper and can be drawn over the fingers, and thus prevent any possibility of infection.

*Dr. Werner.*—I would like to ask the essayist, without bringing up too much talk over the amalgam filling question, whether, in his opinion, he feels that tobacco is an irritant to these ulcers, and also whether he thinks that an amalgam filling would be more of an irritant than any other filling to syphilitic persons?

*Dr. Southwick.*—Tobacco is an irritant to a syphilitic ulcer. An amalgam filling should not irritate more than any other. It is purely a case of mechanical irritation, and does not depend on the components of the filling.

*Dr. Fillebrown.*—They might acquire it a second time.

*Dr. Southwick.*—A person who has had syphilis cannot become infected again.

*Dr. Werner.*—Not even by direct contact?

*Dr. Southwick.*—No, sir. Syphilis may recur after an apparent cure, but it is not due to reinfection.

*Dr. Taft.*—What did you mean, then, when you said that you treated a patient nine times for it?

*Dr. Southwick.*—It was simply one of those cases that was not cured; the disease recurred. It is very difficult to guarantee a cure.

*Dr. Werner.*—Would you expect any beneficial effect from it because it had mercury in it?

*Dr. Southwick.*—No, sir. There wouldn't be enough in it to be of any use.

*President Cooke.*—If the gentlemen have nothing further to say, I will ask Dr. Southwick if he cares to close the subject.

*Dr. Southwick.*—Some of the doctors apparently have not understood what was meant by the term "inunction" as applied in the treatment of syphilis. A simple gray ointment is made of equal parts of mercury and lanolin. In hospitals each patient is given about a drachm and a half at a time, and one syphilitic rubs the other; first, that amount is rubbed in both extremities below the knees; the next day the same amount is rubbed into the thighs; the following day the same amount is rubbed into the back of the body; the next day, into the forearms; and the next day the front part of the body; then the following day a hot bath is given, as hot as the patient can bear it. The day after that the inunction is repeated, going through the same routine. The usual course of treatment is fifteen or twenty inunctions, and in addition to this, chlorate of potassium, two and one-half drachms in a pint of water, is prescribed and used freely, as a gargle, several times a day, and the patient watched for any manifestations of drug action. That is the usual course of treatment after the appearance of the secondary manifestations, when we feel sure that we know what we have to deal with. In certain cases, in addition to this treatment, the iodide of potassium is given internally.

*Dr. Werner.*—I move that we pass the subject with a vote of thanks to the essayist.

Unanimously voted.

*President Cooke.*—Have the gentlemen any specimens to present?

*Mr. Geo. T. Baker.*—I want to call attention to a few experiments that I have been making in regard to rubber dam. I thought I would put some in water for the purpose of preserving it, and to keep it from drying. I found that after a few days it commenced to change color, getting lighter and lighter until finally it became almost white. So I took some different kinds of dam and put them in pure water and found that some of them changed while others did not. The bleached rubber seems to be of material advantage in reflecting the light into inaccessible cavities, approximal cavities, and with patients with small mouths; and on dark days in winter I find it makes quite a difference. It will bleach if left covered with water for about two weeks, and I found that I could bleach it in about fifteen minutes by boiling. I had some that came from the S. S. White Company and some from Hale's on School Street, but neither of those specimens bleached out. The specimens that I got from McDonald, from Bailey, on Boylston Street, and from the American Hard Rubber Company, New York, bleached out all right. Some specimens won't bleach out, apparently, either by boiling or remaining in water.

*Dr. Stoddard.*—Perhaps the reason was that some of the specimens may have been acid-cured.

*Dr. Baker.*—As near as I can make out, the acid-cured is the kind that bleaches, and the sulphur-cured does not.

*Dr. Brackett.*—It was suggested some years ago by Dr. Buckland, of Woonsocket, that the immersion of rubber in water was a means of preserving its elasticity, the idea being identical with that to which Dr. Baker has just referred, with the further recommendation, that after drying it should be closely rolled and protected from the air. I tried immersion in one or two instances, and I found that after an interval the rubber had become very much deteriorated and the water extremely foul. The water was probably not pure in the first place. The whole combination, rubber and water, was very bad, so that I gave up the experiment.

*Dr. Taft.*—There is one thing I have noticed about rubber dam when soaking or washing it. If I put a piece of it in the water of my Boston office, I find, after drying, that instead of the smooth, velvety feeling it has when coming direct from the dealers, there is a wet and sticky feeling to it. But if I take a piece from the same roll and soak it in water in Cambridge, for example, where I live, I find, after drying, that its original smooth and velvety condition is restored; so that I attribute this to some peculiar condition of the water.

*Dr. Eames.*—I have experienced that same thing, but I have attributed it to the difference in the rubber, and not to the difference in the water, and I have removed the difficulty by simply using whiting or soapstone over the rubber to make it smooth.

*Dr. Stevens.*—I want to say just a word as a hint to the preserving of the rubber dam. The way in which I have kept mine is to roll it up, place it in a tin box, and put it in the safe, where it is cool and dark and away from the air, and I find it just as good in a year as it was the day I put it in. I have kept it that way for a good many years.

HARRY E. CUTTER, D.D.S.,
*Editor American Academy of Dental Science.*

